# Hemolytic–Uremic Syndrome in a Grandmother

**DOI:** 10.3201/eid1611.AD1611

**Published:** 2010-11

**Authors:** Lane C. Crawford, Mark L. Crawford, Sean R. Moore

**Affiliations:** Author affiliations: Vanderbilt University School of Medicine, Nashville, Tennessee, USA (L.C. Crawford);; Jackson Purchase Medical Center, Mayfield, Kentucky, USA (M.L. Crawford);; Cincinnati Children’s Hospital Medical Center, Cincinnati, Ohio, USA (S.R. Moore)

**Keywords:** Shiga-toxigenic Escherichia coli, Escherichia coli O157, Shiga toxins, hemolytic–uremic syndrome, clinical laboratory and immunoenzyme techniques, culture media, Tennessee, bacteria, colectomy, another dimension

In the spring of 2007, just weeks before my college graduation, my parents called to tell me that my grandmother was sick. I was worried to hear that she was in the hospital, but like the rest of my family, I had little doubt that she’d be better in no time. At a spry 82 years, my grandmother was the picture of health. She was a nonsmoker, a nondrinker, and had had no medical problems except for mild asthma. A world traveler, avid gardener, and savvy businesswoman, she has been known to do her grocery shopping, attend a bank board meeting, talk her way out of a speeding ticket, and rearrange the living room furniture all in time to prepare dinner for 12 and cut fresh roses for the table. As a loving wife, mother of 5, and grandmother of 10, she is the family matriarch and holds us together in a way I never fully appreciated until that familiar structure was suddenly threatened.

My grandmother’s illness began with acute onset of abdominal cramps and watery, nonbloody diarrhea followed by nausea and vomiting. She had no fever. The diarrhea continued intermittently through the first day and night of her illness, and by the second day, when the diarrhea became grossly bloody, her internist had her admitted to a community hospital.

At the time of admission, she was normotensive and still afebrile. Physical examination findings were unremarkable except for mild bilateral lower abdominal tenderness. The leukocyte count was mildly elevated at 13,500 cells/μL, hemoglobin was within normal limits at 15.0 g/dL, and the platelet count was 263,000 cells/μL. Electrolytes were within normal limits, blood urea nitrogen was mildly elevated at 19 mg/dL, and creatinine was within normal limits at 0.7 mg/dL. Urinalysis findings were also within normal limits. A radiograph of her abdomen showed no signs of a perforated organ. Gastroenterologists were consulted. Their assessment suggested infectious diarrhea as the most likely diagnosis, followed by ischemic colitis and diverticular disease. To check for enteric infections, they ordered stool culture, stool leukocyte count, and a stool *Clostridium difficile* toxin test. Supportive treatment of intravenous hydration and antiemetics was initiated.

On my grandmother’s third day of illness, the bloody diarrhea worsened and the leukocyte count increased to 18,100 cells/μL. A stool sample contained no *C. difficile* toxin but did contain leukocytes. A contrast computed tomography (CT) scan of the abdomen showed pancolitis with 2-cm wall edema and narrowing of the colon lumen. The CT findings of colitis distributed throughout multiple vessels, sparing of the terminal ileum, and good contrast flow in the major mesenteric vessels made a diagnosis of ischemic colitis much less likely; therefore, bacterial pancolitis became the leading diagnosis. Because of increasingly severe bloody diarrhea and leukocytosis, intravenous ciprofloxacin and metronidazole were started empirically while stool culture results were pending.

Over the next 2 days, the bloody diarrhea abated, but abdominal distention and a mild tremor developed. An abdominal radiograph showed air distending the stomach and small bowel. The leukocyte count rose to 30,500 cells/μL, and the platelet count dropped to 105,000 cells/μL. Low urine output, blood urea nitrogen level of 57 mg/dL, and creatinine level of 1.9 mg/dL suggested acute kidney injury, for which increased intravenous fluids and a diuretic were ordered.

None of these developments were overly alarming; nevertheless, my grandmother looked sicker, and my family’s concern deepened. My father (M.L.C.), an orthopedic surgeon, was growing increasingly uneasy, but because hemorrhagic colitis was not his area of expertise, he was content to defer to his colleagues’ judgment. However, as a physician and son, he felt compelled to fully understand his mother’s illness. He began by searching the literature on infectious hemorrhagic colitis and learned that the major bacterial causes are *Shigella, Salmonella, Yersinia, Vibrio, Escherichia coli, Campylobacter,* and *Clostridium* spp (*1*). He then visited the hospital’s bacteriology laboratory, where a technician explained the lab’s stool culture protocol.

My father learned that stool culture on commonly used culture media is quite effective for identification of *Salmonella* spp. and other bacteria not normally found in human bowel. However, because *E. coli* is part of normal bowel flora, a separate strategy is needed to distinguish diarrheagenic *E. coli* serotypes from their nonpathogenic brethren. To do this, most laboratories use a selective medium called sorbitol-MacConkey agar (SMAC) to identify the pathogenic serotype *E. coli* O157:H7. Whereas most other *E. coli* serotypes ferment sorbitol and grow as pink colonies, this serotype does not ferment sorbitol and grows as colorless colonies. When my father asked how often stool culture results from patients with actual bacterial colitis were negative, the technician assured him, “almost never.” Thus, when my grandmother’s stool culture came back negative, my father did not question it.

On my grandmother’s fifth day of illness, colonoscopy showed severe nodular and granular inflammation from rectum to cecum. The gastroenterologist excluded ischemic colitis but, given the negative culture and the colonoscopic appearance, had to consider the possibility of inflammatory bowel disease. Although new onset of this disease was unlikely in an 82-year-old patient, this possibility was covered by prescribing a full dose of steroids; ciprofloxacin was continued. In the meantime, my father continued his literature search, which reinforced his feeling that inflammatory bowel disease was unlikely.

When my father refocused his search on infectious causes of hemorrhagic colitis, *E. coli* emerged as the most frequently described culprit and seemed consistent with my grandmother’s case. He further learned that the serotypes of *E. coli* that cause diarrheal illness in humans do so by producing Shiga toxin and are thus referred to as Shiga toxin–producing *E. coli* (STEC); that the O157:H7 serotype is the most frequently identified type of STEC in the United States; and that it is the most likely serotype to cause hemolytic–uremic syndrome (HUS), a life-threatening condition characterized by hemolytic anemia, low platelet count, and renal failure. He also came across several articles that gave him pause. One recent article stated that 20%–50% of all STEC infections in the United States are caused by non-O157 STEC serotypes, some of which can cause HUS (*2*). A second article explained that non-O157 STEC is not detected by SMAC because, like nonpathogenic *E. coli*, it ferments sorbitol. Instead, both O157 and non-O157 STEC could be detected by enzyme immunoassay for Shiga toxin (*3*). My grandmother’s negative stool culture now seemed less conclusive.

The next morning, the sixth day of illness, my grandmother was transferred to an intensive care unit because of worsening renal failure. Her mental status had declined and her tremor had worsened. My father suspected HUS and conveyed his concern to her internist, who agreed that HUS was a possibility. My father then returned to the laboratory and inquired about the Shiga toxin assay he’d read about. The technician told him that they had recently opted not to buy the toxin assay because of its expense and their satisfaction with SMAC; however, the toxin assay was available at their reference laboratory. Unfortunately, the original stool sample had been discarded. A new specimen was collected, sent, and had negative results for Shiga toxin, but it also grew no gram-negative bacteria on culture. Three days of antimicrobial drugs had effectively sterilized the colon, rendering a false-negative result more likely. That afternoon, the hemoglobin level decreased to 12.0 g/dL, platelet count fell to 71,000 cells/μL, and lactate dehydrogenase level was markedly elevated, all of which could be consistent with developing HUS. Although no schistocytes were evident on peripheral blood smear, the internist was concerned and consulted a nephrologist at a nearby regional hospital. Upon hearing of suspected HUS, he recommended immediately transferring my grandmother for plasmapheresis and possible dialysis.

The next 12 hours were a blur of confusion and frustration for my family. When care of a patient is transferred from one medical team to another, treatment plans often change abruptly, sometimes because of differences in clinical judgment and other times because of imperfect communication between the teams. Whatever the reason, my grandmother left her local hospital with a plan for plasmapheresis and supportive care for HUS, and 1 hour later, after evaluation at the regional hospital, received a diagnosis of acute abdomen and sepsis. A general surgeon was consulted and recommended emergency total colectomy. Without it, he said, she would be dead within the hour. My family was shocked and distressed by this drastic change of plans. My father was particularly hesitant. In his reading about the recommended therapy for STEC and HUS, he had not come across any mention of the need for surgical intervention. Nevertheless, the general surgeon was adamant in his recommendation, and considering the surgical adage “You can never go wrong by looking,” my father advised my grandfather to consent to the operation.

Four hours later, the surgeon returned and explained to the family that the operation had gone well. He had removed all but the distal 12 inches of the colon, which was not as severely affected. He expected that my grandmother would need mechanical ventilation, at least overnight and possibly for 1–2 days. The colon had been reddened and inflamed, but he had found no dead bowel and no perforations. He had noted 1-cm wall edema. Recalling the 2-cm wall edema on the CT image from 3 days ago, my father concluded that the colon had been recovering and regretted his decision to allow the operation.

In the week after my grandmother’s operation, my family learned everything they could about STEC. After learning that it is most frequently transmitted through undercooked beef or contaminated produce, they analyzed their food history. This prompted the discovery of “The Meatloaf,” now infamous in our family lore. Three days before becoming ill, my grandmother had cooked a meatloaf for dinner, which a family member recalled being a bit pink in the middle. Over the next 4 days, all 3 other family members who had eaten the meatloaf experienced mild nonbloody diarrhea but recovered fully. My 4-year-old cousin, who had refused to eat any meatloaf, showed no signs of illness. For once we were grateful for his picky eating. The ground beef in the meatloaf had come from 2 calves from the family farm; the calves had been processed at a local slaughterhouse. Another piece of the puzzle had fallen into place.

Meanwhile, my grandmother continued to need mechanical ventilation and became comatose and anuric. She was evaluated by physicians from the departments of general surgery, infectious disease, nephrology, hematology, pulmonology, and pathology. Despite my father’s suggestions regarding STEC and HUS, their various assessments of her condition culminated in a diagnosis of ischemic colitis with sepsis resulting in acute renal failure, disseminated intravascular coagulation (DIC), and hypotensive brain injury, from which she was not likely to recover.

Each of these diagnoses was far more common than STEC/HUS, but several things simply didn’t fit. First, the operative findings were inconsistent with ischemic colitis. Second, my grandmother lacked the fever and hypotension characteristic of sepsis. Her blood pressure had never dropped to an extent that would be expected to cause severe end-organ damage. Third, the leukocyte count remained markedly elevated despite removal of the suspected source of infection, a battery of potent antimicrobial drugs, and negative blood and urine cultures. The elevated leukocyte count did not fit with sepsis but might have represented acute inflammation associated with HUS (*4*). Finally, although hemoglobin level and platelet count remained low, other coagulation study results were all within normal limits, more consistent with HUS than DIC.

My family remained convinced of the STEC/HUS diagnosis. On postoperative day 6, my father requested that antimicrobial drugs be stopped. If she really had sepsis, she was dying from it in spite of them. If not, they were only obscuring the true diagnosis. The next day my father called the foodborne diseases division of the Centers for Disease Control and Prevention (CDC). He described my grandmother’s case to the physician on call, who agreed, without reservation, with the diagnosis of STEC infection. He explained that sepsis and DIC rarely occurred with STEC infection and that surgery was almost never required. Antimicrobial drugs were not recommended and might even hasten progression to HUS. Although not of proven effectiveness, plasmapheresis was commonly used for treatment of HUS. With this further support of the diagnosis, my father again presented his case to my grandmother’s physicians, and her nephrologist agreed to start a trial of daily plasmapheresis.

Over the next 4 days my grandmother remained comatose, but her urine output slowly began to increase. A nephrologist at a university medical center was consulted and recommended continuing plasmapheresis. That afternoon, my grandmother opened her eyes. By the end of the day—the 16th day of illness—she was recognizing family members, following simple commands, and mouthing words. She could turn her head to look at my grandfather, who was promising her everything in the world. As a combat pilot in World War II, he’d seen his share of battles but none like this. After weeks of feeling lost, helpless, and unable to save his war-time bride, he now regained hope. My family’s relief was indescribable.

Histopathology slides of my grandmother’s colon were sent to a university medical center for review, where pathologists diagnosed acute hemorrhagic necrotizing colitis consistent with STEC infection. Three days after waking from the coma, my grandmother was transferred to that medical center for the remainder of her recovery—incidentally, the same university I was attending as an undergraduate. On the morning of commencement, I was able to visit her wearing my cap and gown. After 11 days in the university hospital and 18 days in a rehabilitation facility, she was finally discharged to go home. Exceeding all expectations, her kidneys and neurologic system recovered fully, and 15 months later her colostomy bag was removed. She is now back to doing all her usual activities—serving as bank director, babysitting her 2 youngest grandchildren, flying to Hawaii for vacation, and cooking for a crowd. Just not meatloaf.

As illustrated by this case report, STEC infection may be difficult to recognize clinically, so appropriate laboratory testing is crucial for accurate and timely diagnosis. Up to 20%–50% of STEC infections in the United States, or ≈37,000 cases per year, are caused by non-O157 *E. coli* serotypes (*2**,**5*), some of which have been associated with severe disease and HUS (*6**,**7*). Failure to test for these serotypes leads to underdiagnosis and underreporting of STEC infection, to the detriment of patient care and public health surveillance, respectively. A 2006 report strongly urges clinical diagnostic laboratories to assay all stool specimens for Shiga toxin, to simultaneously culture the specimens on SMAC for organism isolation, and to forward positive specimens to a public health laboratory (*8*). Because data with regard to implementation of these recommendations are lacking, we surveyed diagnostic laboratories in Tennessee about their protocols for STEC detection and identified factors influencing their choice of protocol.

From July through October 2008, we contacted all clinical laboratories licensed to perform microbiologic or bacteriologic testing in Tennessee and conducted telephone and email interviews with supervisors. This survey was approved by our university’s institutional review board.

From 130 laboratories, we received 117 responses, a high response rate of 90%. Of these 117 respondents, 57 (49%) performed stool cultures in house. Of these 57 laboratories, 46 (81%) included STEC in their routine testing for enteric pathogens, 8 (14%) tested for STEC only in bloody specimens or only with a physician’s order, and 3 (5%) did not test for STEC at all. We further asked the 54 laboratories that tested any or all specimens for STEC about their STEC detection protocol. We found that 38 (70%) of 54 used SMAC alone; 4 (7%) of 54 used Shiga toxin assay alone; and 12 (22%) of 54 used both. Only 8 (15%) of the 54 laboratories used both tests concurrently, and 6 (11%) did so for all specimens as recommended by CDC in 2006.

The second part of our survey aimed to ascertain which factors influenced adherence to the 2006 CDC recommendations. In the 38 laboratories that used SMAC alone, 13 (35%) supervisors stated that they were not familiar with the Shiga toxin assay, and 24 (63%) claimed some familiarity. These 24 were asked to identify reasons for choosing SMAC over Shiga toxin assay. Of the 24, a total of 20 (83%) thought that SMAC alone provides adequate STEC detection, 18 (75%) said SMAC was a better fit for their laboratory’s workflow and staffing situation, 17 (71%) said that they used SMAC because of its lower cost, and 12 (50%) said that they used SMAC because laboratory personnel were unfamiliar with the Shiga toxin assay.

Our survey suggests that Tennessee laboratories fall short of best practice recommendations for detection of non-O157 STEC; only 11% had fully implemented the CDC-recommended protocols. The key factors associated with nonadherence were lack of familiarity with Shiga toxin assays and limited knowledge of current recommendations.

Although laboratories operate under the constraints of third-party reimbursement, local economics, and institutional policy, knowledge of best practice recommendations is essential for making an informed choice of protocol. As evidenced in my grandmother’s case, appropriate laboratory practices must be complemented by physicians’ high index of suspicion and familiarity with diagnostic techniques. Ultimately, it is the treating physician’s responsibility to ensure that all necessary diagnostic tests are ordered, whether in house or at a reference laboratory. The 2006 recommendations were recently emphasized in a 2009 MMWR Recommendations and Reports article (*9*). It is our hope that this recent publication will further contribute to the improved diagnosis of STEC infections, to the benefit of public health, patients, and grandmothers.
